# Normal Incident Long Wave Infrared Quantum Dash Quantum Cascade Photodetector

**DOI:** 10.1186/s11671-016-1611-6

**Published:** 2016-09-09

**Authors:** Feng-Jiao Wang, Fei Ren, Shu-Man Liu, Ning Zhuo, Shen-Qiang Zhai, Jun-Qi Liu, Feng-Qi Liu, Zhan-Guo Wang

**Affiliations:** Key Laboratory of Semiconductor Materials Science, Institute of Semiconductors, Chinese Academy of Sciences, University of Chinese Academy of Sciences and, Beijing Key Laboratory of Low Dimensional Semiconductor Materials and Devices, P.O. Box 912, Beijing, 100083 People’s Republic of China

**Keywords:** Quantum dash, Quantum cascade, Infrared detector

## Abstract

We demonstrate a quantum dash quantum cascade photodetector (QDash-QCD) by incorporating self-assembled InAs quantum dashes into the active region of a long wave infrared QCD. Sensitive photoresponse to normal incident light at 10 μm was observed, which is attributed to the intersubband (ISB) transitions in the quantum well/quantum dash (QW/QDash) hybrid absorption region and the following transfer of excited electrons on the extraction stair-like quantum levels separated by LO-phonon energy. The high density InAs quantum dashes were formed in the Stranski-Krastanow mode and stair-like levels were formed by a lattice matched InGaAs/InAlAs superlattice. A stable responsivity from 5 mA/W at 77 K to 3 mA/W at as high as 190 K was observed, which makes the QDash-QCD promising in high temperature operation.

## Background

Quantum cascade photodetector is one kind of ISB photodetectors based on electrons’ transitions between quantized subbands in the conduction band of semiconductor heterostructures. As a photovoltaic detector, quantum cascade photodetector (QCD) works without an external bias voltage due to asymmetric conduction band profile. This asymmetry is derived from the stair-like subbands separated by LO-phonon energy by choosing appropriate layer thicknesses of the superlattice in the extraction region. This design guarantees a negligible dark current, which makes QCDs promising in large focal plan array and small pixel applications [1, 2]. QCDs have been studied extensively from short wavelength to THz wavelength through the entire infrared spectrum [3–10]. However, the absorption of normal incident light was limited by polarization selection rule for ISB transitions in quantum wells, which restricts the possible applications of QCDs. This leads to strong interest in exploring the possibility of using the intersubband transitions in quantum dot (QD) [11–13], quantum wire [14, 15], and also the dot-in-a-well structure [16, 17] instead of in a QW in terms of polarization relaxation. Effective results have been achieved, because the in-plane confinement of the carriers allows the absorption of photons at normal incidence. Nevertheless, these devices sensitive to normal incident light usually work in photoconductive scheme. The strong dark current derived from photoconductive working scheme seems to be an unavoidable weakness. Lately, quantum dot quantum cascade detectors (QD-QCDs) were demonstrated on both GaAs-based [18] and InP-based [19] material system. The hybrid QW/QD absorption region and the stairs-like extraction region allow the detector to respond to normal incident light and work in photovoltaic scheme at the same time.

Inspired by the concept of QD-QCD, we incorporated quantum dashes into the absorption well of a long-wave infrared photodetector (LWIR) QCD [6] to form the QDash-QCD. In this letter, the high density InAs dashes were formed in the Stranski-Krastanow mode on unstrained InAlAs layer. This device shares advantages of low dark current and semi-3D confinement derived from quantum dashes [20, 21]. Operating with zero bias, the device responded to normal incident radiation with negligible dark current.

## Methods

**Fig. 1:**
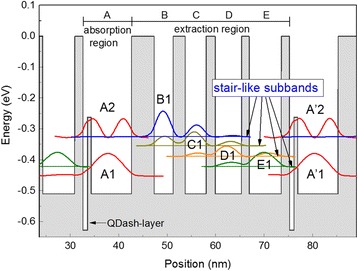
Energy band scheme of one period of the QDash-QCD

The QDash-QCD structures were grown by molecular beam epitaxy on semi-insulating InP (001) substrates. Nineteen periods of the active region—consisting of a 10-nm-wide QW/QDash hybrid region followed by an extraction In_0.52_Al_0.48_As/In_0.53_Ga_0.47_As chirped superlattice—were inserted between a 500-nm-thick n-doped (1 × 10^18^ cm^−3^) In_0.53_Ga_0.47_As bottom contact layer and a 300-nm-thick n-In_0.53_Ga_0.47_As top contact layer. The active region has two components: the active infrared absorption hybrid region and the extraction region, which can be seen in Fig. 1. The absorption hybrid region A consisted of an InAs quantum dash layer and an InGaAs quantum well layer with a thin GaAs barrier layer between them. This region was n-doped with Si to about 4 × 10^17^ cm^−3^. The following extraction region from B to E was formed by a chirped In_0.53_Ga_0.47_As/In_0.52_Al_0.48_As superlattice. The thickness of the layer sequence of a whole period starting from the QDash-layer was as follows (in angstroms): 9(QDash)/8(GaAs)/83(QW)/**47**/39/**25**/43/**19**/54/**16**/66/**17**, with InAlAs layers in bold and InGaAs layers in regular. A control QW-QCD structure with a 10-nm InGaAs quantum well instead of the hybrid region and 30 periods of the active region was also grown. After growth, QDash- and QW-quantum cascade detectors with mesa of 200 μm × 200 μm were fabricated by a standard photolithography, wet chemical etching, metal deposition, and lift-off process.

**Fig. 2:**
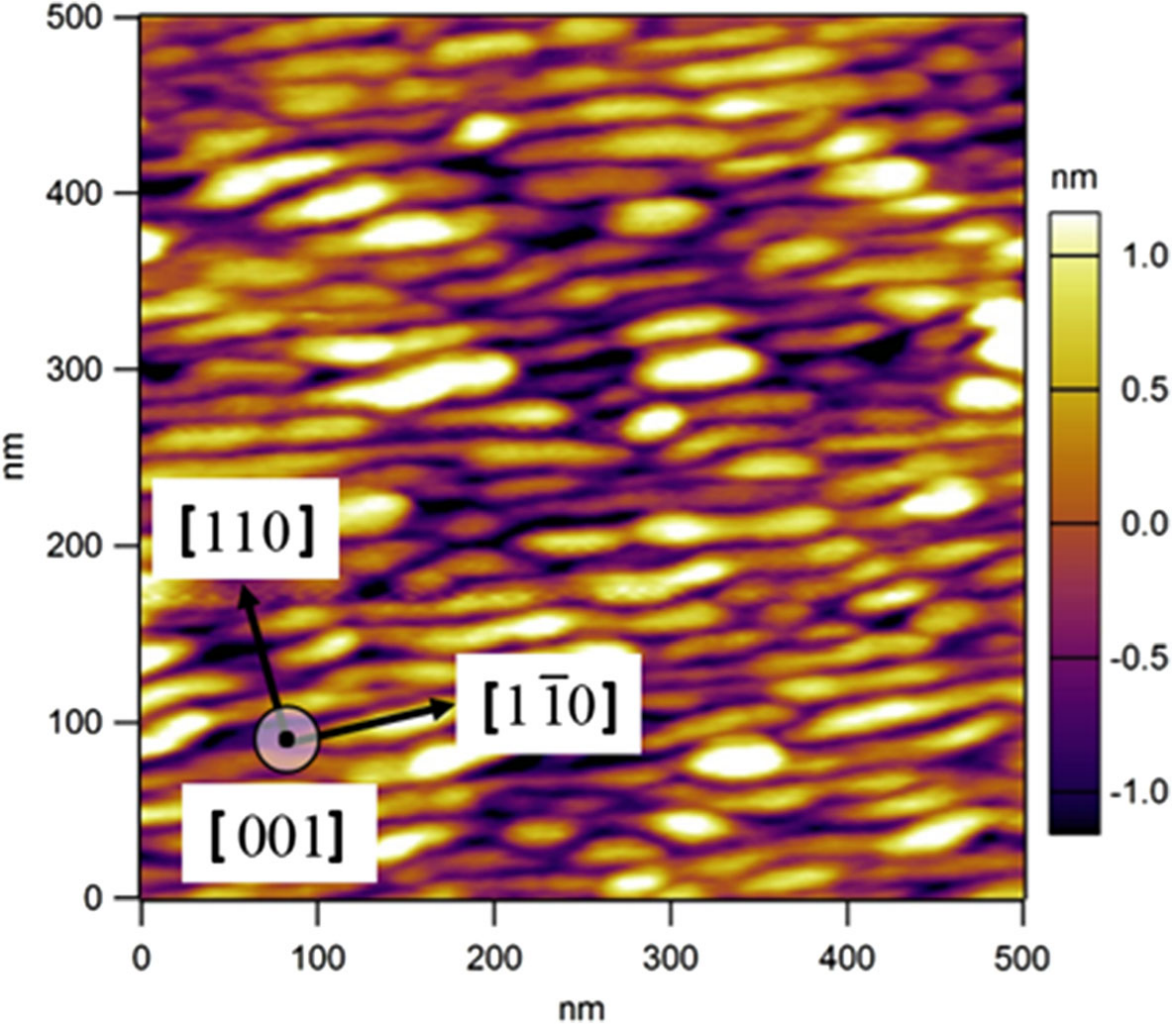
AFM of an uncapped InAs self-assembled QDashes layer on top of the QDash-QCD structure

The InAs QDashes were obtained self-assembly based on the Stranski-Krastanow epitaxial growth mode and the nominal growth rate is about 0.4 ML/s. After the quantum dash layer was deposited, 20 s of ripening time was given under As_4_ protection. The InAs QDash in this study is a kind of elongated nanostructure, as depicted in Fig. 2, whose cross section is similar to that of a quantum dot presented in ref. [22] with dimensions in [110] axis of ~17 nm and [001] axis of ~2.3 nm. The dimension in [$1\overline{1}0$] axis is about a hundred of nanometers in average. Figure 1 shows an atomic force microscopy (AFM) image of a non-overgrown sample with QDashes layer on top of the device structure, where QDashes assemble in a rather dense and parallel manner.

**Fig. 3:**
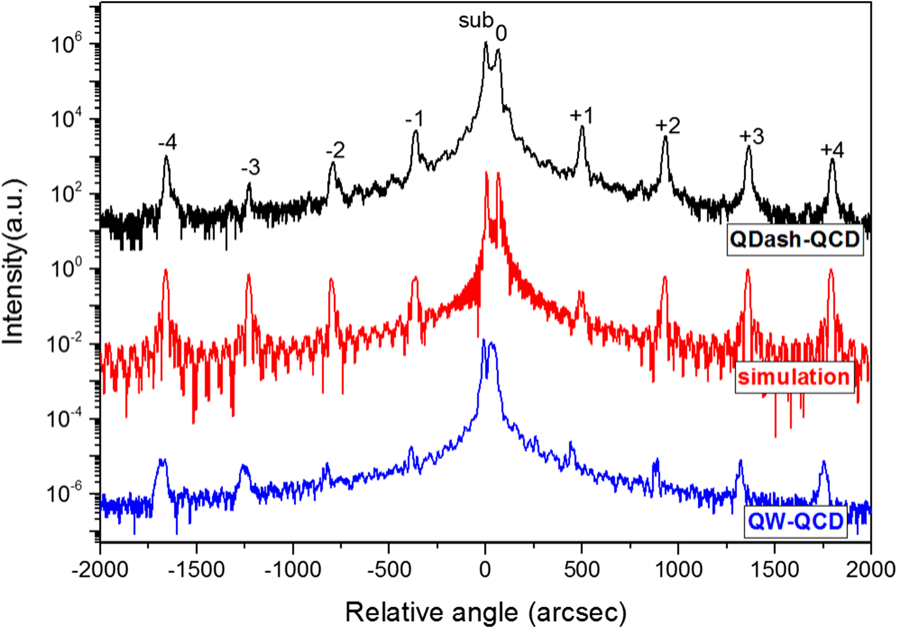
XRD curves of the measured and simulated QDash-QCD structure along with the XRD curve of a QW-QCD structure

Measured and simulated x-ray diffraction rocking curves of QDash-QCD are shown in Fig. 3 and the measured QW-QCD curve is also shown. As can be seen from Fig. 3, the dynamic simulation curve matches the experimental curve well, showing a good control over the growth parameters across the entire epitaxy sequence. Besides, the incorporation of QDashes did not weaken the quality of the superlattice. The clear satellite peaks with good periodicity and narrow linewidths (full width of half maximum ~40 arcsecs) demonstrate a high interfacial quality.

## Results and Discussion

Conduction energy band scheme of one period of QDash-QCD at null bias is shown in Fig. 1. The computation was based on a simplified model by solving one-dimensional Schrödinger equation under envelope-function approximation without considering the quantum confinement of QDashes in the growth plane. The ground-state energy of the QW/QDash hybrid region was determined from the photoluminescence measurements. The InAs layer was simplified into a quantum well whose thickness was adjusted to match the measured ground-state energy. The calculation includes the energy dependence of the effective mass and the effect on the band offset of the strain of the InAs layer with respect to the InP substrate. The active absorption region is a “W-shaped” hybrid structure, consisting of InAs (QDash)/GaAs/InGaAs(QW). The dominant transition is between the hybrid levels A1 and A2, similar to the QW/QD mixed mode reported in ref. [18, 19, 23, 24], leading to a detection wavelength of 10 μm. To ensure an efficient escape process from the absorption region to the cascade extraction region and, at the same time, a considerable resistance to suppress dark current, a resonantly tunneling process was designed between level A2 and level B1. Once the carriers tunnel to level B1, they will transfer through a set of quantum stairs (from B1 to E1) separated by LO phonon energy rapidly in the cascade region. Finally, the excited electrons are transferred from level E1 toward the fundamental level A’1 of the next absorption region by emission of a LO phonon.

**Fig. 4:**
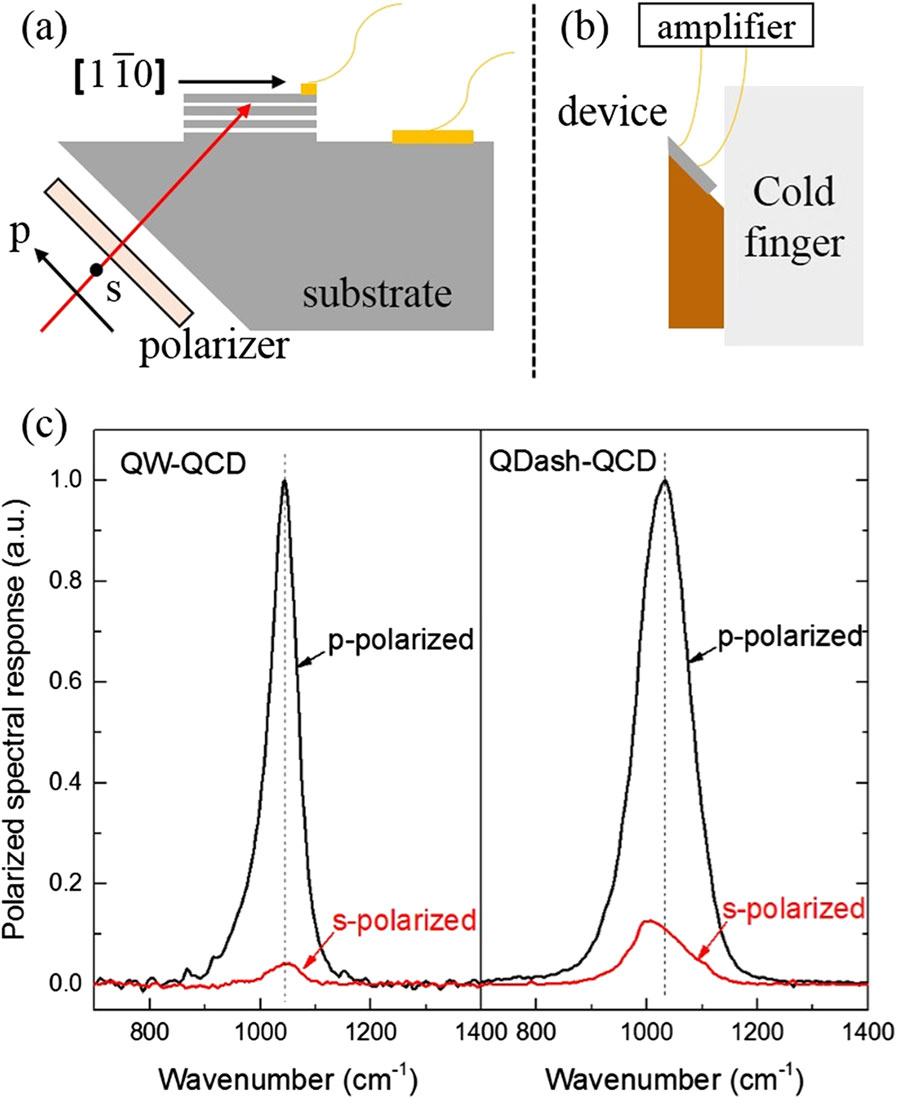
**a** The schematic diagram of the polarized spectral response measurements. **b** The schematic diagram of the 45° facet configuration experimental setup in a cryostat. **c** The s- and p-polarized infrared spectral responses of the QDash-QCD and the control QW-QCD

**Fig. 5:**
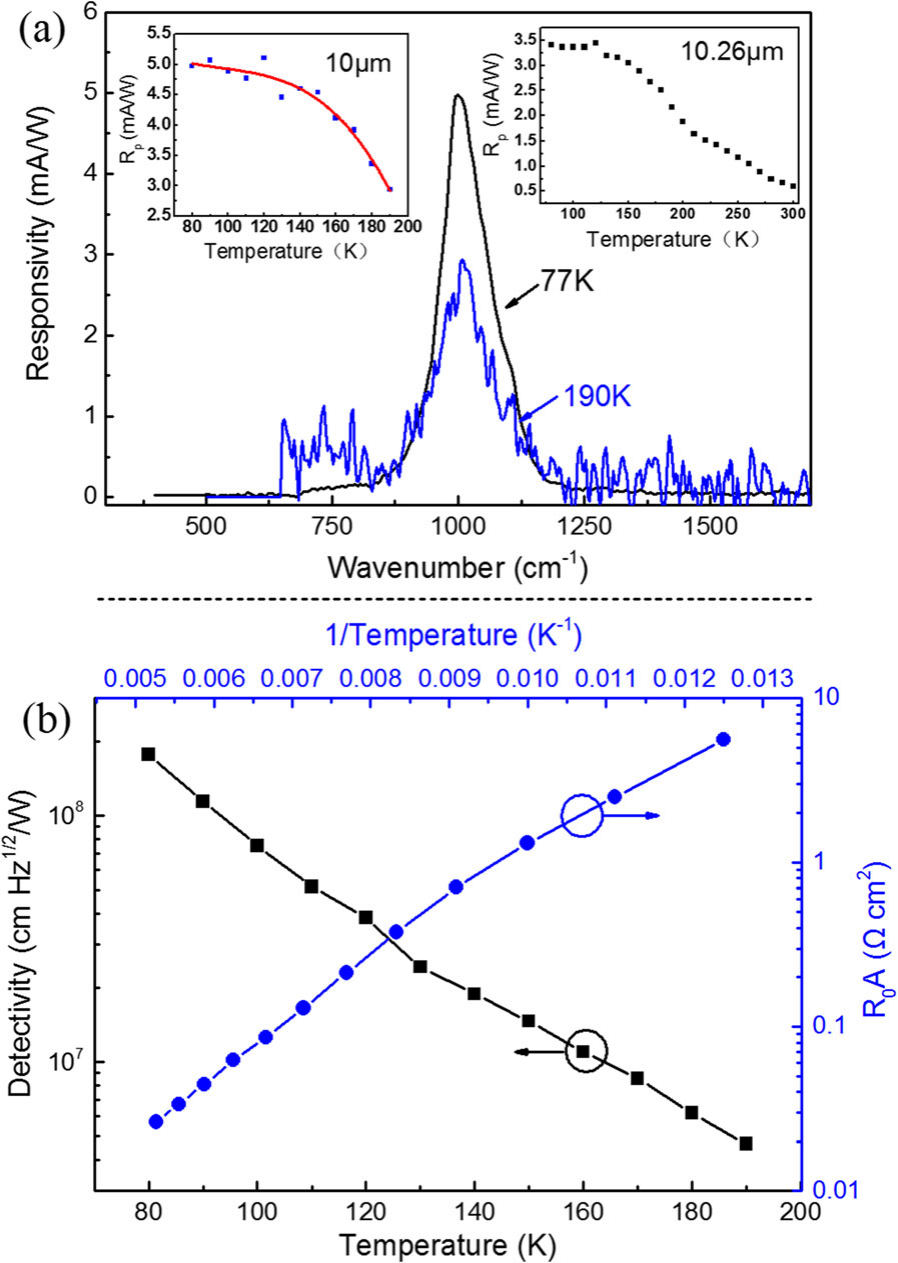
**a** Response spectra of the QDash-QCD at 77 and 190 K. *Left inset*: responsivity of the QDash-QCD from 77 to 190 K. *Right inset*: responsivity of the QDash-QCD at 10.26 μm from 77 to 300 K in every 10 K. **b** Dependence of detectivity and *R*_0_ *A* (product of resistance at zero bias by area of the mesa) of the QDash-QCD on temperature

To figure out the impact of InAs QDashes on the device, we measured the polarization dependent response. Figure 4a shows the schematic diagram of the polarized spectral response measurements. The infrared light incoming vertically to the 45° polished facet of the substrate. When the polarization of the incident infrared light is s-polarized, the E-field is paralleled with the growth plane in [110] direction. While in the case of p-polarized light, the E-field has a 50 % component along the growth direction i.e., in [001] direction and the other 50 % component is paralleled with the growth plane in [$1\overline{1}0$] direction. Figure 4b shows the 45° facet configuration experimental setup in a cryostat. The whole device was mounted on a 45° facet holder which adhered to the cold finger of a cryostat. Figure 4c shows the dependence of spectral response on polarization. The polarized response spectra were measured through a Nicolet 8700 Fourier transform infrared spectrometer (FTIR) in 45° configuration under p-polarized and s-polarized light, respectively. As a control sample, a QW-QCD was also measured. It is to be noted that, a red shift of s-polarized response (1002 cm^−1^) of the QDash-QCD compared to the p-polarized response (1029 cm^−1^) was observed. That is because the dimension of a dash in [$1\overline{1}0$] axis is about a hundred of nanometers which possesses weaker quantum confinement, in the case of p-polarized light, the component of the E-field in this direction can hardly induce ISB transitions. The dominant ISB transitions are derived from the other 50 % E-field component in [001] direction due to the quantum confinement deriving from the QDash and QW at the same time. While in the case of s-polarized light, the E-field is in the [110] direction, the quantum confinement originating from the QDash can overcome the limitation of the polarization selection rule and induce ISB transitions. That is to say, in the case of s-polarized response, the QW/QDash hybrid subbands participating in the transitions are more QDash-like than in p-polarized light. The incorporation of QDashes into the quantum well leads to the downward shift of both the ground-state A1 and the excited state A2 and a tiny decrease of the spacing between these two states, which leads to a redshift of the response spectrum under s-polarized light. The peak of the s-polarized response spectrum lies exactly in the normal response peak position, showing that the QDashes worked dominantly for normal incident absorption, which can be seen in Fig. 5a. Besides, the broadening of the spectra compared to the control sample shows obvious evidence of QDashes due to the size distribution. What is more, the enhancement of s/p ratio from 4.2 to 12.6 % also indicates that the incorporation of quantum dashes into the quantum well enhances normal incidence absorption. The enhanced s/p ratio of 12.6 % is comparable to the traditional GaAs-based quantum dot infrared photodetector (QDIP) whose s/p ratio is about 13 % [25].

Normal incidence spectral reponse were also measured by a Nicolet 8700 FTIR, and the responsivity were calibrated by a circular polarization CO_2_ laser with the wavelength of 10.26 μm. Figure 5a shows the spectra measured at 77 and 190 K at zero bias voltage, the main peak of the photoresponse lies in 10 μm within the atmosphere window of 8–12 μm. The left inset shows the responsivity of the QDash-QCD versus temperature from 77 to 190 K. The QDash-QCD shows a stable responsivity from 5 mA/W at 77 K to 3 mA/W at 190 K, decreased by 40 %. As a comparison, a decrease of 83 % from 28 mA/W at 77 K to 4.2 mA/W at 190 K was achieved in a control QW-QCD with the same doping. This characteristic is similar to the QD-QCD in ref. [22], the incorporation of QDs into the QCD leading to a more stable responsivity than the control sample but with a lower value. That is because the photoresponse is based on the ISB transitions deriving from the hybrid region, the quantum confinement of the nanostructures in plane leads to the stable responsivity. The factors that leads to the low responsivity such as size dispersion of the nanostructure is now under study. To improve the responsivity of QDash-QCD, we may improve the doping density as what we did in ref. [22]. However, for a QCD, the Johnson noise limited detectivity can be obtained by

1 $$ {D}_{\mathrm{J}}^{*}={R}_{\mathrm{p}}\sqrt{\frac{R_0A}{4{k}_{\mathrm{B}}T}}, $$

where *R*_p_ is peak responsivity, *R*_0_*A* is the product of resistance at zero bias by area of the mesa, *k*_B_ is the Boltzmann constant, and *T* is temperature. As to optimizing the performance, it is necessary to have a high spectral responsivity and a high resistance at the same time. But, increasing the doping density in a LWIR detectors seems to produce an unavoidable noise. In this condition, improving the quantum confinement of the nanostructures to obtain an enhanced s/p ratio is an efficient way to improve the responsivity rather than increasing the doping density. We can improve the quantum confinement of the nanostructures from semi-3D confinement of QDashes to 3D confinement of QDs or even submonolayers of QDs to further improve the quantum confinement[25], only if we can design a smart growing method like in ref. [19, 24]. The right inset of Fig. 5a shows the response of the QDash-QCD at 10.26 μm from 77 K to 300 K. A responsivity of 0.59 mA/W was observed at 300 K, indicating the probability of uncooled operation. In addition to the main peak lying at 1022 cm^−1^, a side peak lying at 750 cm^−1^ was observed. It is originated from the transitions from level A1 to level C1 in the extraction region and this is also a thermally excited channel which can be deduced from the dark current measurement.

Dark currents at different temperatures were measured using a keithley 2635B sourcemeter through pulsed current measurement mode and the sample was optically and thermally shielded. The current density at zero bias is about 10^−5^ A/cm^2^, which is a desirable value for long wave infrared photodetector. The low dark current originates mainly from its photovoltaic working scheme and partially from the semi-3D confinement of QDashes. *R*_0_*A* (the product of resistance at zero bias by area of the mesa) at different temperatures were obtained from the dark *I*-*V* curves and were plotted in Fig. 5b as a function of inverse of temperature. The active energy of 62 meV was then deduced from the slope of Arrhenius plots of *R*_0_*A* versus reciprocal of temperature. Together with the Fermi energy of Si doping for 21 meV, a leakage current channel was formed between levels A1 and C1 with an energy spacing of 93 meV, which means a lot of carriers participated in this transition and made a contribution to the side peak lying at 750 cm^−1^. The Johnson noise limited detectivity then can be obtained according to Eq (1). Figure 5b shows the detectivity versus temperature from 77 K to 190 K. A detectivity of 2 × 10^8^ cm Hz^1/2^ W^−1^ was achieved at 77 K and this value decreased to 4.6 × 10^6^ cm Hz^1/2^ W^−1^ at 190 K.

## Conclusions

Normal incident response has been demonstrated in the QDash-QCD by incorporating quantum dashes into the absorption region of a LWIR-QCD. With a detection wavelength of 10 μm, the QDash-QCD possessed a detectivity of 2 × 10^8^ cm Hz^1/2^ W^−1^ along with a responsivity of 5 mA/W at liquid nitrogen temperature. It is noteworthy that a stable responsivity compared to the control QW-QCD has been achieved and the QDash-QCD presented here can work up to 190 K, indicating the high temperature operation.

## Abbreviations

QCD: Quantum cascade photodetector
QDash: Quantum dash
ISB: Intersubband
QW: Quantum well
QD: Quantum dot
LO: Longitudinal optical
3D: Three dimension
ML: Monolayer
AFM: Atomic force microscopy
FTIR: Fourier transform infrared spectrometer
QDIP: Quantum dot infrared photodetector
*R*_0_*A*: The product of resistance at zero bias by area of the mesa
LWIR: Long-wave infrared

## References

[1] Gendron L, Koeniguer C, Berger V, Marcadet X (2005). High resistance narrow band quantum cascade photodetectors. Appl Phys Lett..

[2] Harrer A, Schwarz B, Schuler S, Reininger P, Wirthmuller A, Detz H (2016). 4.3 mum quantum cascade detector in pixel configuration. Opt Express..

[3] Andresen BF, Buffaz A, Carras M, Doyennette L, Nedelcu A, Bois P (2010). State of the art of quantum cascade photodetectors. Proc. SPIE..

[4] Giorgetta FR, Baumann E, Graf M, Yang QK, Manz C, Köhler K (2009). Quantum cascade detectors. IEEE J Quantum Electron..

[5] Sakr S, Giraud E, Dussaigne A, Tchernycheva M, Grandjean N, Julien FH (2012). Two-color GaN/AlGaN quantum cascade detector at short infrared wavelengths of 1 and 1.7μm. Appl Phys Lett.

[6] Graf M, Hoyler N, Giovannini M, Faist J, Hofstetter D (2006). InP-based quantum cascade detectors in the mid-infrared. Appl Phys Lett..

[7] Kong N, Liu JQ, Li L, Liu FQ, Wang LJ, Wang ZG (2010). A 10.7 μm InGaAs/InAlAs quantum cascade detector. Chin Phys Lett.

[8] Giorgetta FR, Baumann E, Graf M, Ajili L, Hoyler N, Giovannini M (2007). 16.5 μm quantum cascade detector using miniband transport. Appl Phys Lett..

[9] Zhai SQ, Liu JQ, Wang XJ, Zhuo N, Liu FQ, Wang ZG (2013). 19 μm quantum cascade infrared photodetectors. Appl Phys Lett..

[10] Graf M, Scalari G, Hofstetter D, Faist J, Beere H, Linfield E (2004). Terahertz range quantum well infrared photodetector. Appl Phys Lett..

[11] Ryzhii V (1996). The theory of quantum-dot infrared phototransistors. Semicond Sci Technol..

[12] Pan D, Towe E, Kennerly S (1998). Normal-incidence intersubband (In, Ga)As/GaAs quantum dot infrared photodetectors. Appl Phys Lett..

[13] Martyniuk P, Rogalski A (2008). Quantum-dot infrared photodetectors: status and outlook. Prog. Quantum Electron..

[14] Tsai CL, Cheng KY, Chou ST, Lin SY (2007). InGaAs quantum wire infrared photodetector. Appl Phys Lett..

[15] Das B, Singaraju P (2005). Novel quantum wire infrared photodetectors. Infrared Physics & Technology..

[16] Krishna S (2005). Quantum dots-in-a-well infrared photodetectors. J Phys D: Appl Phys..

[17] Raghavan S, Rotella P, Stintz A, Fuchs B, Krishna S, Morath C (2002). High-responsivity, normal-incidence long-wave infrared (λ ∼ 7.2 μm) InAs/In 0.15Ga0.85As dots-in-a-well detector. Appl Phys Lett..

[18] Barve AV, Krishna S (2012). Photovoltaic quantum dot quantum cascade infrared photodetector. Appl Phys Lett..

[19] Wang XJ, Zhai SQ, Zhuo N, Liu JQ, Liu FQ, Liu SM (2014). Quantum dot quantum cascade infrared photodetector. Appl Phys Lett..

[20] Liverini V, Bismuto A, Nevou L, Beck M, Gramm F, Müller E (2011). InAs/AlInAs quantum-dash cascade structures with electroluminescence in the mid-infrared. J Cryst Growth..

[21] Miska P, Even J, Platz C, Salem B, Benyattou T, Bru-Chevalier C (2004). Experimental and theoretical investigation of carrier confinement in InAs quantum dashes grown on InP(001). J Appl Phys..

[22] Wang FJ, Zhuo N, Liu SM, Ren F, Ning ZD, Ye XL (2016). Temperature independent infrared responsivity of a quantum dot quantum cascade photodetector. Appl Phys Lett..

[23] Chou ST, Tseng CC, Chen CN, Lin WH, Lin SY, Wu MC (2008). Quantum-dot/quantum-well mixed-mode infrared photodetectors for multicolor detection. Appl Phys Lett..

[24] Zhuo N, Liu FQ, Zhang JC, Wang LJ, Liu JQ, Zhai SQ (2014). Quantum dot cascade laser. Nanoscale Res Lett..

[25] Kim JO, Sengupta S, Barve AV, Sharma YD, Adhikary S, Lee SJ (2013). Multi-stack InAs/InGaAs sub-monolayer quantum dots infrared photodetectors. Appl Phys Lett..

